# Association Between Changes in Heart Rate and Adverse Events in Patients With Non‐Valvular Atrial Fibrillation: A Post Hoc Analysis of the J‐RHYTHM Registry

**DOI:** 10.1002/clc.70122

**Published:** 2025-03-27

**Authors:** Eitaro Kodani, Takeshi Yamashita, Hiroshi Inoue, Hirotsugu Atarashi, Ken Okumura, Hideki Origasa, J‐RHYTHM Registry Investigators

**Affiliations:** ^1^ Department of Cardiovascular Medicine Nippon Medical School Tama Nagayama Hospital Tokyo Japan; ^2^ The Cardiovascular Institute Tokyo Japan; ^3^ Saiseikai Toyama Hospital Toyama Japan; ^4^ Nippon Medical School Tokyo Japan; ^5^ Saiseikai Kumamoto Hospital Kumamoto Japan; ^6^ The Institute of Statistical Mathematics Tokyo Japan

**Keywords:** adverse event, all‐cause death, atrial fibrillation, changing pattern, heart rate

## Abstract

**Background:**

We previously reported that the heart rate (HR) at the time closest to an event or at the last visit during the follow‐up period (HR‐end) was more closely associated with adverse events (AEs) than baseline HR in patients with non‐valvular atrial fibrillation (NVAF). However, it remains uncertain whether changes in HR during the follow‐up period or absolute HR values would be more closely associated with AEs. Thus, we performed post hoc analyses using data from the J‐RHYTHM Registry.

**Methods:**

Of 7406 outpatients with NVAF from 158 institutions, 6886 (age, 69.8 ± 9.9 years; men, 70.8%), who had both baseline HR and HR‐end data, were included. Patients were divided into five groups based on the changing patterns of HR quartiles from baseline (< 63, 63–70, 71–79, and ≥ 80 beats per minute) to the end of follow‐up (no‐change, down‐to‐the‐lowest‐quartile, down‐to‐the‐2nd or ‐3rd‐quartile, up‐to‐the‐2nd or ‐3rd‐quartile, and up‐to‐the‐highest‐quartile).

**Results:**

Hazard ratios for AEs were significantly higher only in the up‐to‐highest‐quartile group (2.89 [95% confidence interval, 1.71–4.90] for thromboembolism, 2.46 [1.53–3.95] for major hemorrhage, and 2.36 [1.51–3.70] for all‐cause death) compared with the no‐change group, after adjusting for confounding factors. Furthermore, in the no‐change group, hazard ratios for AEs were significantly higher in the highest‐to‐highest‐quartile subgroup (5.55 [1.49–20.77] for major hemorrhage and 3.60 [1.03–12.53] for all‐cause death) compared with the 2nd‐to‐2nd‐quartile subgroup.

**Conclusions:**

Both excessive increases in HR and consistently high HR were independently associated with AEs in patients with NVAF. By contrast, modest decrease in HR during follow‐up was associated with lower mortality. Accordingly, it is important to pay attention to changes in HR during follow‐up for the management of patients with AF.

**Clinical Trial Registration:**

The J‐RHYTHM Registry is registered in the University Hospital Medicine Information Network (UMIN) Clinical Trials Registry (unique identifier: UMIN000001569) http://www.umin.ac.jp/ctr/.

## Graphical abstract

Excessive increases in heart rate (HR) and consistently high HR during follow‐up were independently associated with adverse events in patients with atrial fibrillation (AF); whereas, a modest decrease in HR was associated with lower mortality in those with a higher baseline HR. Changes in HR is important for AF management.

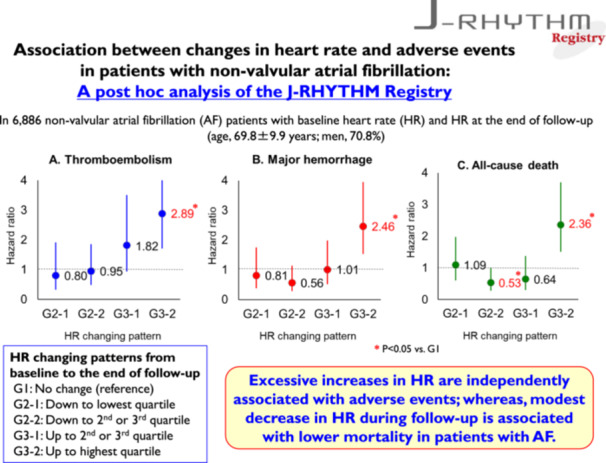

## 1 Introduction

Atrial fibrillation (AF) is a common arrhythmia and a major risk factor for ischemic stroke and heart failure [1, 2]. Heart rate (HR) is reportedly associated with cardiovascular mortality in the general population [3] and in patients with AF [4]. Uncontrolled HR can worsen patient symptoms, quality of life (QOL), and exercise tolerance, and exacerbate heart failure in AF patients. However, a report from the Rate Control Efficacy in Permanent Atrial Fibrillation II (RACE II) trial [5] found that lenient HR control (resting HR < 110 beats per minute [bpm]) was not inferior to strict HR control (resting HR < 80 bpm and HR during moderate exercise < 110 bpm) in terms of cardiovascular morbidity and mortality. As a result, lenient HR control has become an acceptable initial approach for AF management, regardless of heart failure status, according to the 2020 European Society of Cardiology Guidelines [6]. The Japanese guidelines also emphasize the importance of adjusting the HR appropriately while considering subjective symptoms and QOL in each patient [7].

In a previous study, we found that the HR at the time closest to an event or at the last visit during the follow‐up period (HR‐end) was more strongly associated with the incidence of adverse events than the baseline HR in patients with non‐valvular AF (NVAF) [8]. Specifically, the highest quartile of HR‐end (≥ 80 bpm) was independently associated with the incidence of major hemorrhage, all‐cause death, and cardiovascular death compared with the second quartile (64–71 bpm), even after adjusting for known confounding factors and HR‐controlling drug use [8]. In contrast, in a sub‐cohort study of the All Nippon AF In the Elderly (ANAFIE) Registry [9], a baseline HR ≥ 110 bpm was significantly associated with a higher incidence of cardiac events compared with a baseline HR of 60–79 bpm in older NVAF patients aged ≥ 75 years. Nevertheless, it remains uncertain whether changes in HR during the follow‐up period versus absolute HR values would be more strongly associated with adverse events. Therefore, we conducted post hoc analyses to investigate this further using data from the J‐RHYTHM Registry [10].

## 2 Methods

### 2.1 Study Design of the J‐RHYTHM Registry

The J‐RHYTHM Registry was a nationwide prospective observational study that aimed to the status of anticoagulation therapy with warfarin and determined the optimal anticoagulation therapy for Japanese patients with AF [10]. The study design and baseline patient characteristics have been reported elsewhere [10, 11]. The study protocol adhered to the Declaration of Helsinki and was approved by the ethics committee of each participating institution. Written informed consent was obtained from all participants at the time of enrollment. A consecutive series of outpatients with AF of any type were enrolled from 158 institutions between January and July 2009, regardless of their use of anticoagulants, antiarrhythmic drugs, and HR‐controlling drugs. The treating physicians had a decision in selecting the drugs and dosages. Patients with valvular AF (mechanical heart valve and mitral stenosis) were excluded from the subanalysis. HR and blood pressure (BP) were measured at baseline and at each visit during the 2‐year follow‐up period or until an event occurred. Patients with NVAF who had both baseline HR and HR‐end were included in this post hoc analysis.

### 2.2 Follow‐Up and Definition of Endpoints

Patients were followed up for 2 years or until an event occurred, whichever came first. The primary endpoints were as follows: thromboembolism including symptomatic ischemic stroke, transient ischemic attack (TIA), and systemic embolic events; major hemorrhage including intracranial, gastrointestinal, and other hemorrhages requiring hospitalization; and all‐cause and cardiovascular deaths. The diagnostic criteria for each event have been described elsewhere [10, 11].

### 2.3 Patient Grouping

Patients were divided into three groups based on the changing patterns of HR quartiles from baseline (< 63, 63–70, 71–79, and ≥ 80 bpm) to the end of the study (Group 1: no change; Group 2: changed to lower quartiles; and Group 3: changed to higher quartiles) (Figure S1). In the changed groups (Groups 2 and 3), patients were further divided into 4 subgroups (Group 2–1: down to the lowest quartile; Group 2–2: down to the 2nd or 3rd quartiles; Group 3–1: up to the 2nd or 3rd quartiles; and Group 3–2: up to the highest quartile) (Figure S1). In the no‐change group (Group 1), patients were divided into four subgroups (Group 1–1: lowest to the lowest quartile; Group 1–2: 2nd to the 2nd quartile; Group 1–3: 3rd to the 3rd quartile; and Group 1–4: highest to the highest quartile) (Figure S1). In addition, patients were recategorized based on ΔHR (= HR‐end – baseline HR) quartiles (Q1: < −7 bpm; Q2: −7 to −1 bpm: Q3, 0 to 7 bpm: and Q4: ≥ 8 bpm).

### 2.4 Statistical Analysis

Data were presented as mean ± standard deviation (SD) or numbers (percentage). Student's *t*‐test or chi‐square tests were used to compare parameters between two groups, as appropriate. For the comparison of patient characteristics and 2‐year event rates among three or more groups, one‐way analysis of variance and chi‐square tests were used for continuous variables and categorical variables, respectively. Cox proportional hazard models were used for univariable and multivariable analyses to investigate the impact of HR changing patterns on adverse events. Hazard ratios and 95% confidence intervals (CIs) were used to express the results. The Cox proportional hazards assumption was verified using log–log survival curves in the study outcomes. Explanatory variables for the multivariable analysis were adopted from well‐known risk factors used in our previous subanalyses [8, 12]. The initial adjusting model included components of the CHA_2_DS_2_‐VASc score (congestive heart failure, hypertension, age ≥ 75 years, diabetes mellitus, history of stroke or TIA, vascular disease [coronary artery disease], age 65–74 years, female sex) [13], warfarin and antiplatelet use, AF type, and baseline systolic BP and HR (Model 1). The extended adjusting model included all variables from Model 1 plus creatinine clearance (CrCl), hemoglobin level, and β‐blocker, K channel blocker, Ca channel blocker, and digitalis use (Model 2), based on our previous subanalyses [8, 14, 15]. The same analyses were performed to clarify the impact of ΔHR on adverse events. A two‐tailed *P*‐value of less than 0.05 was considered statistically significant. All statistical analyses were performed using SPSS software (version 23.0; IBM Corporation, Armonk, NY, USA).

## 3 Results

Of the 7937 patients with AF enrolled in the J‐RHYTHM Registry, 421 (5.3%) with valvular AF were excluded, and 110 (1.5%) were lost to follow‐up. Of the remaining 7406 patients with NVAF, 520 (7.0%) who had HR measured less than two times during the follow‐up period were excluded. Therefore, this subanalysis included 6886 patients (mean age: 69.8 ± 9.9 years; 70.8% men).

### 3.1 Patient Characteristics and Medications

Table 1 listed the clinical characteristics and medications of the overall cohort of 6886 patients and the three groups (Groups 1, 2, and 3). HR was measured an average of 14.4 ± 5.2 times during the follow‐up period. Baseline HR and HR‐end values were 72.5 ± 13.3 bpm and 73.3 ± 13.3 bpm, respectively. The numbers of patients across baseline HR quartiles (< 63, 63–70, 71–79, and ≥ 80 bpm) were 1621, 1739, 1631, and 1895, respectively. Significant differences were noted in the distribution of AF type, baseline HR, HR‐end, systolic and diastolic BP, CrCl, and hemoglobin levels among the three groups. Tables S1 and S2 show the clinical characteristics and medications of the subgroups within the changed groups (Groups 2–1, 2–2, 3–1, and 3–2) and the no‐change group (Groups 1–1, 1–2, 1–3, and 1–4), respectively. Significant differences were observed in thromboembolic risk (CHADS_2_ and CHA_2_DS_2_‐VASc scores) and bleeding risk (HAS‐BLED score) among the subgroups.

**Table 1:** Patient characteristics and medications by changing patterns of heart rate quartiles.

		Group 1	Group 2	Group 3	
	Overall	No change	Changed to lower quartiles	Changed to higher quartiles	*p*‐value*
Number of patients	6886	2783	1984	2119	
Age, years	69.8 ± 9.9	69.8 ± 10.0	68.5 ± 9.8	69.9 ± 10.0	0.481
Sex, men	4874 (70.8)	1963 (70.5)	1419 (71.5)	1492 (70.4)	0.688
Body mass index, kg/m^2^ (*n* = 5979)	23.6 ± 4.0	23.7 ± 4.5	23.7 ± 3.5	23.6 ± 3.7	0.510
Type of atrial fibrillation					
Paroxysmal	2653 (38.5)	1065 (38.3)	683 (34.4)	905 (42.7)	< 0.001
Persistent	1012 (14.7)	395 (14.2)	338 (17.0)	279 (13.2)	
Permanent	3221 (46.8)	1323 (47.5)	963 (48.5)	935 (44.1)	
Comorbidities					
Coronary artery disease	724 (10.5)	274 (9.8)	211 (10.6)	239 (11.3)	0.263
Cardiomyopathy	573 (8.3)	233 (8.4)	156 (7.9)	184 (8.7)	0.631
HCM	233 (3.4)	89 (3.2)	65 (3.3)	79 (3.7)	0.568
DCM	340 (4.9)	144 (5.2)	91 (4.6)	105 (5.0)	0.652
Congenital heart disease	90 (1.3)	40 (1.4)	27 (1.4)	23 (1.1)	0.544
COPD	123 (1.8)	52 (1.9)	41 (2.1)	30 (1.4)	0.265
Hyperthyroidism	120 (1.7)	53 (1.9)	39 (2.0)	28 (1.3)	0.202
Risk factors for stroke					
Heart failure	1794 (27.5)	783 (28.1)	531 (26.8)	580 (27.4)	0.571
Hypertension	4176 (60.6)	1695 (60.9)	1175 (59.2)	1306 (61.6)	0.269
Age (≥ 75 years)	2387 (34.7)	973 (35.0)	658 (33.2)	756 (35.7)	0.219
Diabetes mellitus	1265 (18.4)	523 (18.8)	355 (17.9)	387 (18.3)	0.723
Stroke/TIA	940 (13.7)	400 (14.4)	264 (13.3)	276 (13.0)	0.344
CHADS_2_ score	1.7 ± 1.2	1.7 ± 1.2	1.6 ± 1.2	1.7 ± 1.2	0.088
CHA_2_DS_2_‐VASc score	2.8 ± 1.6	2.8 ± 1.6	2.7 ± 1.6	2.8 ± 1.6	0.087
HAS‐BLED score (*n* = 6541)	1.5 ± 1.0	1.5 ± 1.0	1.5 ± 1.0	1.5 ± 1.0	0.210
Heart rate measurement times	14.4 ± 5.2	14.5 ± 5.3	14.5 ± 5.0	14.2 ± 5.1	0.104
Baseline heart rate, bpm	72.5 ± 13.3	73.4 ± 14.9	79.6 ± 11.0	64.7 ± 7.7	0.001
Heart rate‐end, bpm	73.3 ± 13.3	73.5 ± 14.7	65.7 ± 7.4	80.1 ± 11.9	< 0.001
Systolic BP, mmHg	126.0 ± 16.1	126.2 ± 16.1	126.5 ± 16.2	125.2 ± 16.2	0.016
Diastolic BP, mmHg	73.5 ± 14.9	73.4 ± 11.0	74.5 ± 10.8	72.4 ± 11.4	< 0.001
CrCl, mL/min (*n* = 5671)	68.4 ± 27.7	68.9 ± 28.7	69.2 ± 26.4	67.0 ± 27.5	0.035
Hemoglobin, g/dL (*n* = 6117)	13.7 ± 1.7	13.7 ± 1.7	13.9 ± 1.7	13.6 ± 1.8	< 0.001
Medications					
Warfarin	5931 (86.1)	2405 (86.4)	1714 (86.4)	1812 (85.5)	0.612
PT‐INR (*n* = 5931)	1.91 ± 0.49	1.92 ± 0.49	1.91 ± 0.52	1.90 ± 0.48	0.582
TTR†, % (*n* = 5611)	59.4 ± 29.1	59.7 ± 29.0	59.5 ± 28.9	58.9 ± 29.4	0.721
Antiplatelet	1810 (26.3)	728 (26.2)	502 (25.3)	580 (27.4)	0.316
Aspirin	1653 (22.7)	626 (22.5)	437 (22.0)	500 (23.6)	0.461
Warfarin + antiplatelet	1258 (18.3)	508 (18.3)	351 (17.7)	399 (18.8)	0.641
ARB/ACE‐I	3663 (53.2)	1462 (52.5)	1060 (53.4)	1141 (53.8)	0.640
Na channel blockers	1413 (20.5)	579 (21.8)	413 (22.0)	421 (21.1)	0.766
β‐blockers	1080 (15.7)	454 (17.1)	302 (16.1)	324 (16.2)	0.610
K channel blockers‡	985 (14.3)	431 (16.2)	270 (14.4)	284 (14.2)	0.103
Ca channel blockers	470 (6.8)	178 (6.7)	141 (7.5)	151 (7.6)	0.434
Digitalis	748 (10.9)	299 (11.2)	217 (11.5)	232 (11.6)	0.913

*Note:* Data are number of patients (%) or mean ± standard deviation.

Abbreviations: HCM, hypertrophic cardiomyopathy; DCM, dilated cardiomyopathy; COPD, chronic obstructive pulmonary disease; TIA, transient ischemic attack; CHADS_2_, congestive heart failure, hypertension, age ≥ 75 years, diabetes mellitus, and history of stroke or TIA; CHA_2_DS_2_‐VASc, additionally, vascular disease (coronary artery disease), age 65–74 years, and female sex; HAS‐BLED, hypertension (systolic BP ≥ 140 mmHg), abnormal renal/liver function, stroke, bleeding history or predisposition, labile INR (episodes of INR ≥ 3.5), elderly (age > 65 years), drugs (use of antiplatelets)/alcohol concomitantly; bpm, beats per minute; heart rate‐end, heart rate at the time closest to an event or at the last visit of follow‐up; BP, blood pressure; CrCl, creatinine clearance; PT‐INR, prothrombin time international normalized ratio; TTR, time in therapeutic range; ARB, angiotensin II receptor blocker; ACE‐I, angiotensin converting enzyme inhibitor.

* Comparison among 3 groups (No change, Changes to the lower quartiles, and Changes to the higher quartiles).

^†^ Target PT‐INR was 2.0–3.0 (< 70 years) or 1.6–2.6 (≥ 70 years) based on Japanese guidelines (Ref [7]).

^‡^ Bepridil was classified as a K channel blocker.

### 3.2 Event Rates

During the 2‐year follow‐up period, thromboembolism, major hemorrhage, all‐cause death, and cardiovascular death occurred in 117 (1.7%), 130 (1.9%), 157 (2.3%), and 58 (0.8%) patients, respectively. The incidence rates of these events were 0.9, 0.9, 1.1, and 0.4 per 100 person‐years, respectively, during a follow‐up period of 13,758 person‐years. Table S3 summarizes the 2‐year event rates. Significant differences were observed in the rates of thromboembolism, major hemorrhage, and all‐cause death among the three groups. Furthermore, all event rates differed significantly among the five groups (no‐change group and four changed subgroups). In the no‐change group, rates of major hemorrhage and all‐cause death were significantly different among the four subgroups.

### 3.3 Influence of HR Changing Patterns on Adverse Events

Changes in HR from baseline to the end of the study, stratified by event types, are summarized in Table S4. The mean HR did not change significantly from baseline to the end of follow‐up in event‐free patients (72.5 ± 13.2 to 72.7 ± 12.6 bpm, *p* = 0.127). However, there was a significant increase in the mean HR patients with any event (73.2 ± 13.9 to 81.8 ± 19.8 bpm, *p* < 0.001). In patients who suffered from thromboembolism, major hemorrhage, all‐cause death, and cardiovascular death, the mean HR‐end increased to 79.8 ± 16.5, 81.6 ± 18.9, 83.3 ± 22.8, and 79.3 ± 20.7 bpm, respectively, from the baseline HR. These HR‐end values were significantly higher compared to event‐free patients (72.7 ± 12.6 bpm) (Table S4). The changes in HR distribution from baseline to the end of follow‐up are shown in Figures S2A–2G, for entire patients (Figure S2A), those with and without any event (Figures S2B and 2C), and by event types (Figures S2D–2G). The HR distribution shifted to the right (indicating a faster HR), especially in patients with any event (Figure S2C).

**Figure 1:**
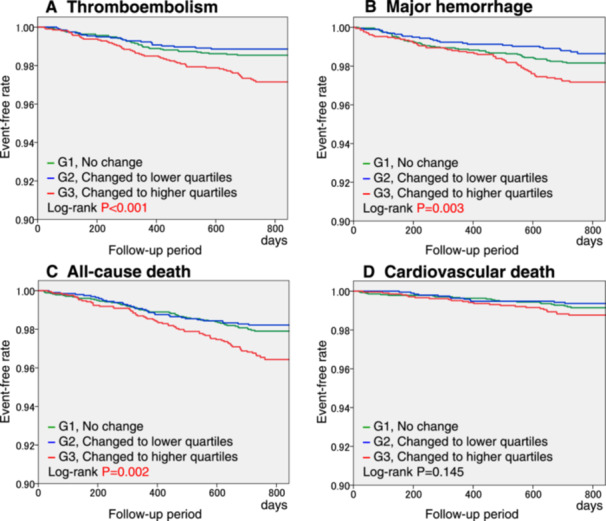
Kaplan–Meier curves for thromboembolism (A), major hemorrhage (B), all‐cause death (C), and cardiovascular death (D) by changing patterns of heart rate quartiles G, group.

In the three groups with HR changing patterns, the hazard ratios for thromboembolism, major hemorrhage, and all‐cause death were significantly higher in Group 3 (changed to higher quartiles) compared to Group 1 (no change), in both the unadjusted and adjusted models (Table 2). The Kaplan–Meier curves for adverse events based on each HR changing pattern are shown in Figure 1. There were significant differences in the event‐free rates of thromboembolism, major hemorrhage, and all‐cause death among the three groups (Figure 1).

**Table 2:** Hazard ratios for events by changing patterns of heart rate quartiles (Cox proportional hazards analysis).

	Thromboembolism		Major hemorrhage		All‐cause death		Cardiovascular death	
	Hazard ratio (95% CI)	*p*‐value	Hazard ratio (95% CI)	*p*‐value	Hazard ratio (95% CI)	*p*‐value	Hazard ratio (95% CI)	*p*‐value
Univariable (unadjusted)								—
G1: No change	Reference	—	Reference	—	Reference	—	Reference	—
G2: Changed to lower quartiles	0.78 (0.47–1.32)	0.784	0.71 (0.44–1.15)	0.168	0.86 (0.56–1.62)	0.483	0.76 (0.37–1.53)	0.437
G3 Changed to higher quartiles	1.91 (1.27–2.87)	0.002	1.52 (1.04–2.23)	0.032	1.65 (1.16–2.35)	0.006	1.46 (0.82–2.60)	0.204
Multivariable (Model 1)								
G1: No change	Reference	—	Reference	—	Reference	—	Reference	—
G2: Changed to lower quartiles	0.77 (0.45–1.30)	0.325	0.70 (0.43–1.14)	0.147	0.80 (0.52–1.23)	0.310	0.74 (0.36–1.52)	0.415
G3: Changed to higher quartiles	2.16 (1.38–3.38)	0.001	1.74 (1.15–2.63)	0.008	1.95 (1.32–2.87)	0.001	1.43 (0.77–2.66)	0.260
Multivariable (Model 2)								
G1: No change	Reference	—	Reference	—	Reference	—	Reference	—
G2: Changed to lower quartiles	0.88 (0.49–1.58)	0.676	0.64 (0.36–1.12)	0.118	0.72 (0.46–1.16)	0.178	0.59 (0.25–1.36)	0.214
G3: Changed to higher quartiles	2.49 (1.50–4.12)	< 0.001	1.84 (1.16–2.92)	0.009	1.62 (1.04–2.53)	0.033	1.08 (0.53–2.21)	0.832

*Note:* Model 1: adjusted for components of CHA_2_DS_2_‐VASc score, warfarin and antiplatelet use, type of atrial fibrillation, and baseline systolic blood pressure and heart rate (*n* = 6886).

Model 2: adjusted for variables of Model 1 plus creatinine clearance, hemoglobin level, and β‐blocker, K channel blocker, Ca channel blocker, and digitalis use (*n* = 5287).

Abbreviations: CI, confidence interval; G, group.

When the changed groups were divided into four subgroups, the hazard ratios for all adverse events were significantly high in Group 3–2 (up to the highest quartile) compared to Group 1 (no change), which served as a reference, in the unadjusted model (Table 3). After fully adjusting for confounding factors (Model 2), the hazard ratios for thromboembolism (2.89 [95% CI, 1.71–4.90]), major hemorrhage (2.46 [1.53–3.95]), and all‐cause death (2.36 [1.51–3.70]) in Group 3–2 remained significantly higher than those in Group 1 (Table 3 and Figures S3A–3C); whereas, the hazard ratio for cardiovascular death (1.54 [0.72–3.27]) in Group 3–2 was no longer significant (Table 3 and Figure S3D). In addition, the hazard ratio for all‐cause death (0.53 [0.28–1.00]) in Group 2–2 (down to the 2nd or 3rd quartile) was significantly lower than that in Group 1 (no change) (Table 3 and Figure S3C).

**Table 3:** Hazard ratios for events by changing patterns of heart rate quartiles in changed groups (Cox proportional hazards analysis).

	Thromboembolism		Major hemorrhage		All‐cause death		Cardiovascular death	
	Hazard ratio (95% CI)	*p*‐value	Hazard ratio (95% CI)	*p*‐value	Hazard ratio (95% CI)	*p*‐value	Hazard ratio (95% CI)	*p*‐value
Univariable (unadjusted)								—
G1: No change	Reference	—	Reference	—	Reference	—	Reference	—
G2‐1: Down to lowest quartile	0.65 (0.29–1.46)	0.300	0.82 (0.43–1.57)	0.546	1.19 (0.70–2.03)	0.522	1.49 (0.68–3.23)	0.317
G2‐2: Down to 2^nd^ or 3^rd^ quartile	0.86 (0.48–1.57)	0.631	0.64 (0.36–1.63)	0.144	0.65 (0.37–1.14)	0.134	0.31 (0.09–1.02)	0.054
G3‐1: Up to 2^nd^ or 3^rd^ quartile	1.44 (0.85–2.42)	0.174	0.73 (0.40–1.32)	0.298	0.56 (0.30–1.04)	0.066	0.70 (0.28–1.72)	0.430
G3‐2: Up to highest quartile	2.42 (1.53–3.83)	< 0.001	2.39 (1.58–3.60)	< 0.001	2.85 (1.96–4.13)	< 0.001	2.29 (1.23–4.27)	0.009
Multivariable (Model 1)								
G1: No change	Reference	—	Reference	—	Reference	—	Reference	—
G2‐1: Down to lowest quartile	0.69 (0.31–1.55)	0.371	0.88 (0.46–1.69)	0.700	1.25 (0.73–2.13)	0.412	1.62 (0.75–3.54)	0.222
G2‐2: Down to 2^nd^ or 3^rd^ quartile	0.82 (0.45–1.51)	0.525	0.62 (0.34–1.15)	0.127	0.58 (0.33–1.03)	0.061	0.28 (0.08–0.95)	0.041
G3‐1: Up to 2^nd^ or 3^rd^ quartile	1.74 (0.98–3.11)	0.061	0.84 (0.46–1.57)	0.580	0.69 (0.36–1.34)	0.276	0.78 (0.30–2.03)	0.609
G3‐2: Up to highest quartile	2.42 (1.50–3.90)	< 0.001	2.45 (1.61–3.74)	< 0.001	2.89 (1.96–4.25)	< 0.001	2.02 (1.06–3.85)	0.033
Multivariable (Model 2)								
G1: No change	Reference	—	Reference	—	Reference	—	Reference	—
G2‐1: Down to lowest quartile	0.80 (0.33–1.92)	0.610	0.81 (0.38–1.75)	0.598	1.09 (0.60–1.98)	0.787	1.19 (0.47–3.01)	0.710
G2‐2: Down to 2^nd^ or 3^rd^ quartile	0.95 (0.49–1.86)	0.889	0.56 (0.28–1.14)	0.111	0.53 (0.28–1.00)	0.049	0.23 (0.05–1.00)	0.051
G3‐1: Up to 2^nd^ or 3^rd^ quartile	1.82 (0.94–3.51)	0.076	1.01 (0.51–1.98)	0.982	0.64 (0.30–1.37)	0.254	0.59 (0.19–1.83)	0.358
G3‐2: Up to highest quartile	2.89 (1.71–4.90)	< 0.001	2.46 (1.53–3.95)	< 0.001	2.36 (1.51–3.70)	< 0.001	1.54 (0.72–3.27)	0.262

*Note:* Variables adjusted for Model 1 (*n* = 6886) and Model 2 (*n* = 5287) are listed in Table 3.

Abbreviations: CI, confidence interval; G, group.

Among four subgroups of the no‐change group, the hazard ratios for major hemorrhage and all‐cause death were significantly higher in Group 1–4 (highest to the highest quartile) compared to Group 1–2 (2nd to the 2nd quartile), which was used as a reference, in the unadjusted model (Table 4). In the fully adjusted model (Model 2), the hazard ratios for thromboembolism were comparable among four groups (Table 4 and Figure S4A). In contrast, the hazard ratios for cardiovascular death (17.04 [1.36–213.20]), in addition to major hemorrhage (5.55 [1.49–20.77]) and all‐cause death (3.60 [1.03–12.53]), in Group 1–4 were significantly higher than those in Group 1–2 (Table 4 and Figures S4B–4D).

**Table 4:** Hazard ratios for events by changing patterns of heart rate quartiles in no‐change groups (Cox proportional hazards analysis).

	Thromboembolism		Major hemorrhage		All‐cause death		Cardiovascular death	
	Hazard ratio (95% CI)	*p*‐value	Hazard ratio (95% CI)	*p*‐value	Hazard ratio (95% CI)	*p*‐value	Hazard ratio (95% CI)	*p*‐value
Univariable (unadjusted)								—
G1‐1: Lowest to lowest quartile	0.58 (0.22–1.52)	0.268	0.83 (0.33–2.09)	0.688	1.06 (0.40–2.85)	0.904	2.48 (0.50–12.27)	0.267
G1‐2: 2^nd^ to 2^nd^ quartile	Reference	—	Reference	—	Reference	—	Reference	—
G1‐3: 3^rd^ to 3^rd^ quartile	0.88 (0.33–2.31)	0.793	0.42 (0.11–1.55)	0.192	1.43 (0.52–3.95)	0.487	2.51 (0.46–13.70)	0.288
G1‐4: Highest to highest quartile	1.05 (0.47–2.33)	0.913	2.17 (1.02–4.59)	0.043	3.11 (1.37–7.05)	0.007	3.51 (0.77–16.04)	0.105
Multivariable (Model 1)								
G1‐1: Lowest to lowest quartile	1.00 (0.35–2.88)	0.994	0.52 (0.18–1.53)	0.236	1.79 (0.63–5.09)	0.279	2.94 (0.50–17.40)	0.236
G1‐2: 2^nd^ to 2^nd^ quartile	Reference	—	Reference	—	Reference	—	Reference	—
G1‐3: 3^rd^ to 3^rd^ quartile	0.67 (0.24–1.84)	0.436	0.55 (0.14–2.14)	0.391	1.60 (0.56–4.54)	0.382	3.23 (0.55–18.97)	0.195
G1‐4: Highest to highest quartile	0.41 (0.11–1.49)	0.175	5.39 (1.64–17.73)	0.006	2.68 (0.88–8.21)	0.084	6.48 (0.82–50.98)	0.076
Multivariable (Model 2)								
G1‐1: Lowest to lowest quartile	0.87 (0.26–2.92)	0.823	0.56 (0.17–1.87)	0.347	2.32 (0.71–7.53)	0.161	4.53 (0.42–43.34)	0.221
G1‐2: 2^nd^ to 2^nd^ quartile	Reference	—	Reference	—	Reference	—	Reference	—
G1‐3: 3^rd^ to 3^rd^ quartile	0.52 (0.16–1.63)	0.259	0.54 (0.11–2.74)	0.457	1.63 (0.45–5.83)	0.457	6.79 (0.65–71.32)	0.111
G1‐4: Highest to highest quartile	0.28 (0.07–1.20)	0.086	5.55 (1.49–20.77)	0.011	3.60 (1.03–12.53)	0.044	17.04 (1.36–213.20)	0.028

*Note:* Variables adjusted for Model 1 (*n* = 6886) and Model 2 (*n* = 5287) are listed in Table 3.

Abbreviations: CI, confidence interval; G, group.

### 3.4 Influence of ΔHR on Adverse Events

Regarding ΔHR quartiles, the hazard ratios for thromboembolism (3.31 [1.66–6.57]), major hemorrhage (2.61 [1.42–4.83]), and all‐cause death (2.32 [1.28–4.21]) were significantly higher in the highest quartile (Q4: ΔHR ≥ 8 bpm) compared to the second quartile (Q2: ΔHR −7 to −1 bpm) in Model 2 (Table S5 and Figures S5A–5C); whereas, the hazard ratio for cardiovascular death (1.77 [0.63–4.99]) in Q4 was not significant (Table S5 and Figure S5D).

## 4 Discussion

The major findings of this study are as follows. First, there was a significant association between the up‐to‐higher‐quartile pattern, particularly the up‐to‐highest‐quartile pattern, and adverse events in patients with NVAF. Second, in the no‐change group, only the highest‐to‐highest‐quartile pattern was associated with adverse events. Third, a moderate increase in HR, defined as ≥ 8 bpm, from baseline was found to be significantly associated with adverse events. These findings are consistent with our previous report [8], which found that higher HR at the end of follow‐up is associated with adverse events. Importantly, a novel finding is that a modest decrease in HR during follow‐up is associated with lower mortality when the baseline HR is in the highest quartile.

### 4.1 HR Control and Changing Patterns in AF Patients

The optimal target HR in patients with AF has long been a subject of debate [4, 16, 17, 18, 19]. It has traditionally been believed that a resting HR of < 80 bpm and an HR during exercise of < 110 (115) bpm are optimal targets for patients with AF. However, this could not be proven in the RACE II trial [5]. As a result, guidelines now accept a lenient HR control as the initial approach and recommend an adjustment of HR as needed to improve patient symptoms and QOL [6, 7]. However, most studies have not considered changes in HR during the follow‐up period. HR can easily fluctuate due to changes in hemodynamics and/or autonomic nerve activity. Therefore, variable HR changing patterns may exist infinitely during the follow‐up period. Our previous study showed that an HR ≥ 80 bpm at the end of follow‐up was independently associated with major hemorrhage, all‐cause death, and cardiovascular death in patients with NVAF, whereas baseline HR was not [8]. Thus, in this analysis, we simplified HR changing patterns using only the baseline HR and HR at the end of follow‐up (HR‐end), resulting in three patterns: no change, changed to lower quartiles, and changed to higher quartiles. Subsequently, we further divided the changed groups and the no‐change group into subgroups to determine whether changes in HR during the follow‐up period or absolute HR values were more closely associated with adverse events.

### 4.2 Impact of HR Changes to Higher Quartiles on Adverse Events

First, we divided patients into three groups based on HR changing patterns from baseline to the end of the follow‐up period. Group 3 (changed to higher quartiles), which consisted of patients with an increase in HR during follow‐up, showed a higher incidence of thromboembolism, major hemorrhage, and all‐cause death compared to Group 1 (no change), even after adjusting for known confounding factors and use of HR‐controlling drugs (Table 2). Furthermore, when we divided the changed groups into four subgroups, the hazard ratios for thromboembolism, major hemorrhage, and all‐cause death in Group 3–2 (up to the highest quartile) were the highest among the four subgroups (Table 3). These findings indicate that increases in HR, particularly an excessive increase in HR up to the highest quartile, are closely associated with the incidence of adverse events in patients with NVAF. Such increases in HR could be an early sign of events or a result of changes in hemodynamics and an increase in sympathetic nerve activity during the follow‐up period. This suggests that an excessive increase in HR could be either the result of nonfatal events such as bleeding or a clinical indication of more severe adverse events. Notably, according to the analyses on ΔHR quartiles (Table S5), an increase in HR ≥ 8 bpm during the follow‐up period was significantly associated with adverse events. Therefore, patients with an increase in HR ≥ 8 bpm should be managed cautiously to avoid subsequent severe adverse events.

### 4.3 Impact of Consistently High HR on Adverse Events

Among the four subgroups of the no‐change group (Group 1), only Group 1–4 (highest to the highest quartile) demonstrated a significant association with an increased incidence of major hemorrhage, all‐cause death, and cardiovascular death (Table 4). This finding suggests that a consistently high HR of ≥ 80 bpm during follow‐up may serve as a marker for subsequent adverse events, even in cases where the patient's HR is stable. Previous studies have also shown that higher HR (> 65 bpm) is associated with increased all‐cause mortality in patients with AF in the Outcomes Registry for Better Informed Treatment of Atrial Fibrillation (ORBIT‐AF) trial [4], and a baseline HR of ≥ 110 bpm is independently linked to adverse events in patients with persistent or permanent AF in the Fushimi AF Registry [20]. In addition, a sub‐cohort analysis of the ANAFIE Registry demonstrated that a baseline HR of ≥ 110 bpm is connected to a higher incidence of cardiac events in AF patients aged ≥ 75 years [9].

Despite recent guidelines accepting lenient HR control in patients with AF [6, 7] based on the findings of the RACE II trial [5], our results support the notion that a target resting HR of < 80 bpm may be more suitable than ≥ 80 bpm to avoid an increased risk of adverse events. Patients with consistently high HR of ≥ 80 bpm during follow‐up may have chronic unfavorable conditions that lead to continuous activation of the sympathetic nervous system. This continuous high HR can negatively impact cardiac function, potentially resulting in tachycardia‐induced cardiomyopathy. Additionally, AF patients with high HR reportedly exhibit increased indices of platelet activity and a prothrombotic state, indicating that strict HR control can improve thrombogenicity and platelet activity [21]. Therefore, careful management of patients with consistently high HR is essential to identify undiagnosed underlying diseases or hidden unfavorable conditions and prevent subsequent adverse events (see below).

### 4.4 Impact of HR Changes to Lower Quartiles on Adverse Events

In the present study, when the groups that experienced changes were divided into four subgroups, Group 2–2 (down to the 2nd or 3rd quartile) showed significantly lower all‐cause mortality compared to Group 1 (no change) (Table 3). This result suggests that a slight decrease in HR to less than 80 bpm, but not an excessive decrease to less than 63 bpm, may have a positive effect on mortality. Similar to tachycardia, an excessively low HR could indicate either nonfatal temporary events or the likelihood of more severe adverse events in the future. While the hazard ratios for all‐cause and cardiovascular deaths in Group 2–1 (down to the lowest quartile) were not statistically significant in this study, the hazard ratios for Group 2–1 were higher than those for Group 2–2 (down to the 2nd or 3rd quartile) and Group 3–1 (up to the 2nd or 3rd quartile). This finding exhibits a J‐shaped curve and aligns with the relationship between HR‐end and cardiovascular death in our previous study [8] and the link between baseline HR and all‐cause mortality in the ORBIT‐AF trial [4]. In addition, previous studies have found that both higher HR [4, 9, 20] and lower HR are independently associated with all‐cause mortality in patients with AF [4, 16].

### 4.5 Limitations

This study has several limitations. First, it was a post hoc analysis of the J‐RHYTHM Registry [11, 22] and therefore serves as a hypothesis‐generating study. However, the causal relationship between changes in HR and adverse events, as well as the underlying mechanisms, could not be determined through this observational study. Second, the study subjects were recruited from only 158 institutions in Japan, and most participating physicians specialized in cardiology and the management of cardiac arrhythmias. As a result, these findings may not be applicable to the entire Japanese population with NVAF. In addition, since all study subjects were Japanese, the results may not be generalized to other racial/ethnic populations. Third, changes in drugs such as oral anticoagulants and HR‐controlling medications, as well as their dosages, and adherence were not taken into account during the follow‐up period. Fourth, the J‐RHYTHM Registry was initiated during the warfarin era, and it remains uncertain whether the present findings can be applied to patients in the era of direct oral anticoagulants. Fifth, the HR was not consistently recorded during AF, particularly in patients with paroxysmal AF. Although this may have affected the overall results of entire patients, we have confirmed, in a previous subanalysis using the same cohort [8], that the type of AF did not impact the outcomes of the entire study cohort. Finally, while we focused on changes in HR fluctuation during the follow‐up period was only assessed through baseline HR and HR‐end. Therefore, all changes in HR during the follow‐up period were not considered in the present analyses.

## 5 Conclusions

Both excessive increases in HR and consistently high HR during follow‐up were found to be independently associated with adverse events in patients with NVAF. For patients with a higher baseline HR, modest decreases in HR during follow‐up could be associated with lower mortality. Accordingly, it is important to pay attention to changes in HR during follow‐up for the management of patients with AF.

## Author Contributions

Dr. Kodani contributed to the statistical analyses and wrote the manuscript. Drs. Yamashita, Inoue, Atarashi, and Okumura are on the Executive Committee of the J‐RHYTHM Registry, and they planned and supervised the study comprehensively and edited the manuscript, as appropriate. Dr. Origasa contributed as a statistical advisor.

## Conflicts of Interest

E.K. received remuneration from Daiichi‐Sankyo; T.Y. received remuneration from Bayer Healthcare, Bristol‐Myers Squibb, Daiichi‐Sankyo, Nippon Boehringer Ingelheim, Novartis, and Otsuka Pharmaceutical; H.I. Inoue received remuneration from Daiichi‐Sankyo; H.A. received remuneration from Daiichi‐Sankyo; K.O. received remuneration from Boehringer Ingelheim, Bristol‐Myers Squibb, Daiichi‐Sankyo, Johnson & Johnson, and Medtronic; and H.O. received remuneration from Johnson & Johnson.

## Supporting information

SupplementaryMaterials_Ver4.

## Data Availability

The data that support the findings of this study are available from the corresponding author upon reasonable request.
