# Supplementary material for: Association Between Changes in Heart Rate and Adverse Events in Patients With Non‐Valvular Atrial Fibrillation: A Post Hoc Analysis of the J‐RHYTHM Registry
Source: Clin Cardiol. 2025 Mar 27;48(4):e70122. doi: 10.1002/clc.70122 (PMC11947616; doi:10.1002/clc.70122)
Supplement: Supplementary file 1 — SupplementaryMaterials_Ver4. [file CLC-48-e70122-s001.pdf]

Supplementary Materials

Association between changes in heart rate and adverse events in patients with non-valvular atrial fibrillation: A post hoc analysis of the J-RHYTHM Registry

Eitaro Kodani, Takeshi Yamashita, Hiroshi Inoue, Hirotsugu Atarashi, Ken Okumura, Hideki Origasa, on behalf of the J-RHYTHM Registry Investigators

1. Table S1. Patient characteristics and medications by changing patterns of heart rate quartiles in changed groups
2. Table S2. Patient characteristics and medications by changing patterns of heart rate quartiles in no-change group
3. Table S3. Two-year event rates by changing patterns of heart rate quartiles
4. Table S4. Changes in heart rate by event types
5. Table S5. Hazard ratios for events by Δheart rate quartiles
6. Figure S1. Changing patterns of heart rate quartiles and grouping
7. Figure S2. Distribution of baseline HR and HR-end by event types
8. Figure S3. Hazard ratios for adverse events by changing patterns of heart rate quartiles in changed groups
9. Figure S4. Hazard ratios for adverse events by changing patterns of heart rate quartiles in no-change groups
10. Figure S5. Hazard ratios for adverse events by Δheart rate quartiles
11. Supplementary Figure Legends

Table S1. Patient characteristics and medications by changing patterns of heart rate quartiles in changed groups

	Changed to lower quartiles		Changed to higher quartiles		
	Group 2-1	Group 2-2	Group 3-1	Group 3-2	
	Down to lowest quartile	Down to 2nd or 3rd quartile	Up to 2nd or 3rd quartile	Up to highest quartile	*P*-value*
Number of patients	757	1227	1085	1034	
Age, years	69.7±9.9	69.5±9.8	69.7±9.5	70.1±10.4	0.581
Sex, men	551 (72.8)	868 (70.7)	779 (71.8)	713 (69.0)	0.439
Body mass index, kg/m^2 (n=5979)	23.6±3.6	23.7±3.4	23.6±3.7	23.5±3.8	0.740
Type of atrial fibrillation					
Paroxysmal	322 (42.5)	361 (29.4)	546 (50.3)	359 (34.7)	
Persistent	116 (15.3)	222 (18.1)	131 (12.1)	148 (14.3)	<0.001
Permanent	319 (42.1)	644 (52.5)	408 (37.8)	527 (51.0)	
Comorbidities					
Coronary artery disease	84 (11.1)	127 (10.4)	129 (11.9)	110 (10.6)	0.430
Cardiomyopathy	60 (7.9)	96 (7.8)	91 (8.4)	93 (9.0)	0.881
HCM	30 (4.0)	35 (2.9)	48 (4.4)	31 (3.0)	0.185
DCM	30 (4.0)	61 (5.0)	43 (4.0)	62 (6.0)	0.163
Congenital heart disease	12 (1.6)	15 (1.2)	17 (1.6)	6 (0.6)	0.224
COPD	12 (1.6)	29 (2.4)	10 (0.9)	20 (1.9)	0.118
Hyperthyroidism	10 (1.3)	29 (2.4)	17 (1.6)	11 (1.1)	0.138
Risk factors for stroke					
Heart failure	186 (24.6)	345 (28.1)	265 (24.4)	315 (30.5)	0.008
Hypertension	451 (59.6)	724 (59.0)	657 (60.6)	649 (62.8)	0.437
Age (≥75 years)	251 (33.2)	407 (33.2)	368 (33.9)	388 (37.5)	0.193
Diabetes mellitus	144 (19.0)	211 (17.2)	193 (17.8)	194 (18.8)	0.731
Stroke/TIA	96 (12.7)	168 (13.7)	137 (12.6)	139 (13.4)	0.585
$CHADS_2$ score	1.6±1.2	1.7±1.2	1.6±1.2	1.8±1.3	0.014
CHA_2DS_2-VASc score	2.7±1.6	2.8±1.6	2.8±1.6	2.9±1.7	0.037
HAS-BLED score (n=6541)	1.4±1.0	1.5±1.0	1.4±1.0	1.6±1.0	0.038
Heart rate measurement times	14.8±5.1	14.2±5.0	14.5±5.1	13.9±5.2	0.002
Baseline heart rate, bpm	73.7±10.1	83.2±9.9	61.1±5.8	68.5±7.7	<0.001
Heart rate-end, bpm	58.0±3.8	70.4±4.4	71.4±4.4	89.2±10.4	<0.001
Systolic BP, mmHg	126.6±16.8	126.5±15.8	125.0±15.6	125.3±16.7	0.073
Diastolic BP, mmHg	73.9±10.7	74.9±10.8	72.0±10.5	72.8±12.3	<0.001
CrCl, mL/min (n=5671)	69.1±26.3	69.3±26.4	68.3±26.9	65.7±28.1	0.032
Hemoglobin, g/dL (n=6117)	13.7±1.7	13.9±1.8	13.5±1.7	13.6±1.9	<0.001
Medications					
Warfarin	640 (84.5)	1074 (87.5)	921 (84.9)	891 (86.2)	0.266
PT-INR (n=5931)	1.90±0.52	1.91±0.52	1.90±0.49	1.90±0.48	0.887
TTR†, % (n=5611)	58.2±28.9	60.2±28.9	59.2±29.3	58.6±29.5	0.639
Antiplatelet	178 (23.5)	324 (26.4)	279 (25.7)	301 (29.1)	0.113
Aspirin	154 (20.3)	283 (23.1)	241 (22.2)	259 (25.0)	0.203
Warfarin+antiplatelet	116 (15.3)	235 (19.2)	186 (17.1)	213 (20.6)	0.045

ARB/ACE-I	404 (53.4)	656 (53.5)	583 (53.7)	558 (54.0)	0.924
Na channel blockers	153 (21.4)	260 (22.3)	200 (19.6)	221 (22.6)	0.501
β-blockers	122 (17.1)	180 (15.5)	170 (16.7)	154 (15.7)	0.709
K channel blockers‡	98 (13.7)	172 (14.8)	156 (15.3)	128 (13.1)	0.144
Ca channel blockers	51 (7.1)	90 (7.7)	70 (6.9)	81 (8.3)	0.494
Digitalis	89 (12.4)	128 (11.0)	112 (11.0)	120 (12.3)	0.750

Data are number of patients (%) or mean±standard deviation

HCM, hypertrophic cardiomyopathy; DCM, dilated cardiomyopathy; COPD, chronic obstructive pulmonary disease; TIA, transient ischemic attack; $CHADS_2$, congestive heart failure, hypertension, age ≥75 years, diabetes mellitus, and history of stroke or TIA; CHA_2DS_2-VASc, additionally, vascular disease (coronary artery disease), age 65–74 years, and female sex; HAS-BLED, hypertension (systolic BP ≥140 mmHg), abnormal renal/liver function, stroke, bleeding history or predisposition, labile INR (episodes of INR ≥3.5), elderly (age >65 years), drugs (use of antiplatelets)/alcohol concomitantly; bpm, beats per minute; heart rate-end, heart rate at the time closest to an event or at the last visit of follow-up; BP, blood pressure; CrCl, creatinine clearance; PT-INR, prothrombin time international normalized ratio; TTR, time in therapeutic range; ARB, angiotensin II receptor blocker; ACE-I, angiotensin converting enzyme inhibitor.

* Comparison among 5 groups (No change group shown in Table 1 and above 4 groups).

† Target PT-INR was 2.0–3.0 (<70 years) or 1.6–2.6 (≥70 years) based on Japanese guidelines (Ref. 7).

‡ Bepridil was classified as a K channel blocker.

Table S2. Patient characteristics and medications by changing patterns of heart rate quartiles in no-change group

	No change in heart rate quartiles				
	**Group 1-1**	**Group 1-2**	**Group 1-3**	**Group 1-4**	
	**Lowest to lowest quartile**	**2nd to 2nd quartile**	**3rd to 3rd quartile**	**Highest to highest quartile**	***P*-value***
Number of patients	753	621	490	919	
Age, years	69.4±9.2	70.4±9.8	69.5±10.5	69.9±10.4	0.293
Sex, men	549 (72.9)	432 (69.6)	342 (69.8)	640 (69.6)	0.423
Body mass index, kg/m^2 (n=2423)	23.7±3.3	23.5±3.5	23.7±3.6	23.8±6.0	0.664
Type of atrial fibrillation					
Paroxysmal	463 (61.5)	279 (44.9)	136 (27.8)	187 (20.3)	
Persistent	88 (11.7)	73 (11.8)	63 (12.9)	171 (18.6)	<0.001
Permanent	202 (26.8)	269 (43.3)	291 (59.4)	561 (61.0)	
Comorbidities					
Coronary artery disease	83 (11.0)	70 (11.3)	47 (9.6)	74 (8.1)	0.113
Cardiomyopathy	60 (8.0)	63 (10.1)	35 (7.1)	75 (8.2)	0.293
HCM	33 (4.4)	23 (3.7)	12 (2.4)	21 (2.3)	0.063
DCM	27 (3.6)	40 (6.4)	23 (4.7)	54 (5.9)	0.070
Congenital heart disease	12 (1.6)	8 (1.3)	7 (1.4)	13 (1.4)	0.972
COPD	10 (1.3)	9 (1.4)	8 (1.6)	25 (2.7)	0.134
Hyperthyroidism	16 (2.1)	9 (1.4)	11 (2.2)	17 (1.8)	0.752
Risk factors for stroke					
Heart failure	170 (22.6)	180 (29.0)	139 (28.4)	294 (32.0)	<0.001
Hypertension	477 (63.3)	366 (58.9)	293 (59.8)	559 (60.8)	0.369
Age (≥75 years)	233 (30.9)	230 (37.0)	164 (33.5)	346 (37.6)	0.019
Diabetes mellitus	120 (15.9)	119 (19.2)	90 (18.4)	194 (21.1)	0.061
Stroke/TIA	111 (14.7)	98 (15.8)	56 (11.4)	135 (14.7)	0.203
$CHADS_2$ score	1.6±1.2	1.8±1.3	1.6±1.2	1.8±1.2	0.005
CHA_2DS_2-VASc score	2.7±1.6	2.9±1.7	2.8±1.6	2.9±1.6	0.027
HAS-BLED score (n=2649)	1.5±1.0	1.5±1.0	1.4±1.0	1.6±1.1	0.021
Heart rate measurement times	14.8±5.3	14.8±5.3	15.3±5.3	13.7±5.3	<0.001
Baseline heart rate, bpm	56.8±5.2	67.6±2.9	74.5±2.4	90.3±10.2	<0.001
Heart rate-end, bpm	57.1±4.7	67.8±2.2	74.7±2.5	90.2±10.4	<0.001
Systolic BP, mmHg	127.3±16.5	124.5±14.8	125.6±15.1	126.8±16.9	0.007
Diastolic BP, mmHg	71.6±10.2	72.1±10.4	74.2±10.5	75.4±11.8	<0.001
CrCl, mL/min (n=2288)	68.8±26.2	67.4±25.5	71.2±30.7	68.8±31.6	0.253
Hemoglobin, g/dL (n=2470)	13.5±1.6	13.5±1.7	13.8±1.6	13.8±1.8	0.004
Medications					
Warfarin	656 (87.1)	523 (84.2)	429 (87.6)	797 (86.7)	0.323
PT-INR (n=2405)	1.91±0.49	1.93±0.46	1.94±0.54	1.90±0.47	0.424
TTR†, % (n=2261)	59.4±29.3	63.2±27.7	59.1±29.8	57.9±29.1	0.016
Antiplatelet	190 (25.2)	165 (26.6)	111 (22.7)	262 (28.5)	0.105
Aspirin	164 (21.8)	152 (24.5)	91 (18.6)	219 (23.8)	0.076
Warfarin+antiplatelet	138 (18.3)	117 (18.8)	81 (16.5)	172 (18.7)	0.740

ARB/ACE-I	415 (55.1)	309 (49.8)	249 (50.8)	489 (53.2)	0.197
Na channel blockers	155 (21.5)	140 (23.4)	89 (19.3)	195 (22.1)	0.456
β-blockers	110 (15.3)	108 (18.1)	89 (19.3)	147 (16.6)	0.279
K channel blockers‡	103 (14.3)	120 (20.1)	81 (17.6)	127 (14.4)	0.010
Ca channel blockers	36 (5.0)	38 (6.4)	35 (7.6)	69 (7.8)	0.121
Digitalis	76 (10.6)	68 (11.4)	53 (11.5)	102 (11.6)	0.925

Data are number of patients (%) or mean±standard deviation

Abbreviations are the same as in Supplementary Table 1.

* Comparison among 4 groups.

† Target PT-INR was 2.0–3.0 (<70 years) or 1.6–2.6 (≥70 years) based on Japanese guidelines (Ref. 7).

‡ Bepridil was classified as a K channel blocker.

Table S3. Two-year event rates by changing patterns of heart rate quartiles

	Number of patients	Thromboembolism	Major hemorrhage	All-cause death	Cardiovascular death
**G1: No change**	2783	39 (1.4%)	49 (1.8%)	55 (2.0%)	22 (0.8%)
**G2: Changed to lower quartiles**	1984	22 (1.1%)	25 (1.3%)	34 (1.7%)	12 (0.6%)
**G3 Changed to higher quartiles**	2119	56 (2.6%)	56 (2.6%)	68 (3.2%)	24 (1.1%)
***P*-value**		<0.001	0.004	0.002	0.168
**Subgroups in changed groups**					
**G2-1: Down to lowest quartile**	757	7 (0.9%)	11 (1.5%)	18 (2.4%)	9 (1.2%)†
**G2-2: Down to 2nd or 3rd quartile**	1227	15 (1.2%)	14 (1.1%)	16 (1.3%)	3 (0.2%)
**G3-1: Up to 2nd or 3rd quartile**	1085	22 (1.1%)	14 (1.3%)	12 (1.1%)	6 (0.6%)
**G3-2: Up to highest quartile**	1034	34 (3.3%)	42 (4.1%)‡	56 (5.4%)‡	18 (1.7%)‡
P*-value		<0.001	<0.001	<0.001	0.002
**Subgroups in no-change groups**					
**G1-1: Lowest to lowest quartile**	753	7 (0.9%)	9 (1.2%)	9 (1.2%)	6 (0.8%)
**G1-2: 2nd to 2nd quartile**	621	10 (1.6%)	9 (1.4%)	7 (1.1%)	2 (0.3%)
**G1-3: 3rd to 3rd quartile**	490	7 (1.4%)	3 (0.6%)	8 (1.6%)	4 (0.8%)
**G1-4: Highest to highest quartile**	919	15 (1.6%)	28 (3.0%)	31 (3.4%)	10 (1.1%)
***P*-value**		0.622	0.003	0.003	0.427

Data are number of patients (%).

G, group.

* Comparison among 5 groups (Group 1 and 4 subgroups).

† P<0.05 versus Group 2-2.

‡ P<0.05 versus Group 3-1.

Table S4. Changes in heart rate by event types

	Number	Baseline heart rate	Heart rate-end	*P*-Value*
**Entire patients**	6886	72.5±13.3	73.3±13.3	<0.001
**Evet free**	6482	72.5±13.2	72.7±12.6	0.127
**Any Event**	404	73.2±13.9	81.8±19.8†	<0.001
**Thromboembolism**	117	72.5±14.0	79.8±16.5†	<0.001
**Major hemorrhage**	130	72.5±13.1	81.6±18.9†	<0.001
**All-cause death**	157	74.2±14.5	83.3±22.8†	<0.001
**Cardiovascular death**	58	72.7±14.9	79.3±20.7†	0.064

Data are mean±standard deviation.

Heart rate-end, heart rate at the time closest to an event or at the last visit of follow-up.

* Comparison between baseline heart rate and heart rate-end by paired-t test.

† P<0.001 versus event free.

Table S5. Hazard ratios for events by Δheart rate quartiles (Cox proportional hazards analysis)

	Thromboembolism		Major hemorrhage		All-cause death		Cardiovascular death	
	**Hazard ratio (95% CI)**	***P*-value**	**Hazard ratio (95% CI)**	***P*-value**	**Hazard ratio (95% CI)**	***P*-value**	**Hazard ratio (95% CI)**	***P*-value**
**Univariable (unadjusted)**								-
**Q1: <-7 bpm**	1.42 (0.73–2.70)	0.311	0.98 (0.51–1.90)	0.960	1.97 (1.10–3.51)	0.022	1.97 (0.76–5.07)	0.161
**Q2: -7 to -1 bpm**	Reference	-	Reference	-	Reference	-	Reference	-
**Q3: 0 to 7 bpm**	1.25 (0.65–2.40)	0.497	1.44 (0.79–2.60)	0.233	1.27 (0.69–2.32)	0.444	1.92 (0.76–4.86)	0.171
**Q4: ≥8 bpm**	2.71 (1.50–4.89)	0.001	2.75 (1.58–4.77)	<0.001	3.27 (1.90–5.62)	<0.001	2.46 (0.99–6.12)	0.054
**Multivariable (Model 1)**								
**Q1: <-7 bpm**	1.33 (0.69–2.56)	0.396	0.96 (0.50–1.86)	0.910	1.90 (1.07–3.40)	0.030	1.85 (0.72–4.78)	0.204
**Q2: -7 to -1 bpm**	Reference	-	Reference	-	Reference	-	Reference	-
**Q3: 0 to 7 bpm**	1.28 (0.67–2.46)	0.452	1.43 (0.79–2.59)	0.241	1.22 (0.67–2.25)	0.517	1.88 (0.74–4.79)	0.185
**Q4: ≥8 bpm**	2.59 (1.43–4.67)	0.002	2.58 (1.49–4.49)	0.001	2.82 (1.63–4.85)	<0.001	2.14 (0.86–5.36)	0.104
**Multivariable (Model 2)**								
**Q1: <-7 bpm**	1.61 (0.76–3.43)	0.216	0.97 (0.46–2.01)	0.925	1.64 (0.87–3.07)	0.126	1.58 (0.55–4.59)	0.399
**Q2: -7 to -1 bpm**	Reference	-	Reference	-	Reference	-	Reference	-
**Q3: 0 to 7 bpm**	1.32 (0.61–2.84)	0.479	1.30 (0.66–2.55)	0.443	1.05 (0.54–2.06)	0.878	1.71 (0.61–4.85)	0.311
**Q4: ≥8 bpm**	3.31 (1.66–6.57)	0.001	2.61 (1.42–4.83)	0.002	2.32 (1.28–4.21)	0.005	1.77 (0.63–4.99)	0.278

CI, confidence interval; Q, quartile; bpm; beats per minute.

Variables adjusted for Model 1 (n=6886) and Model 2 (n=5287) are listed in Table 3.

Figure S1

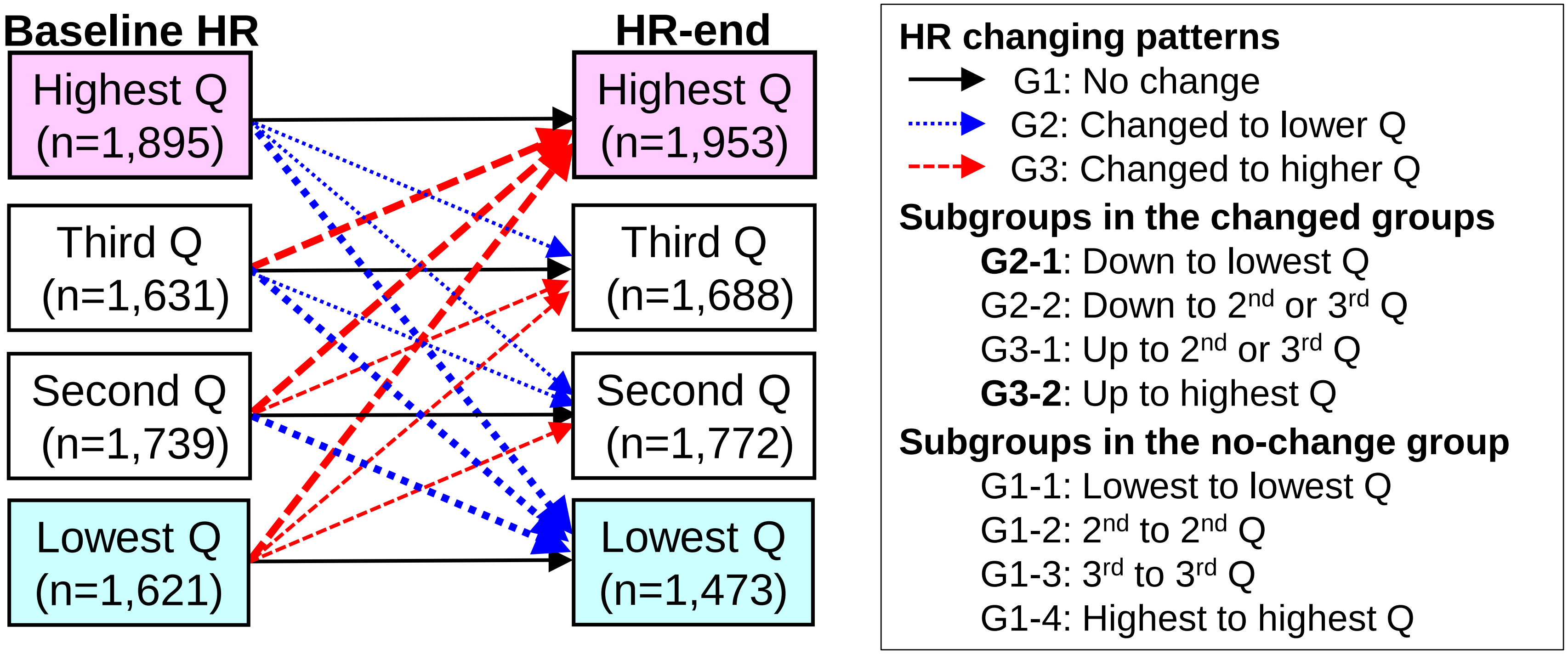
Baseline HR
HR-end
Highest Q (n=1,895)
Third Q (n=1,631)
Second Q (n=1,739)
Lowest Q (n=1,621)
Highest Q (n=1,953)
Third Q (n=1,688)
Second Q (n=1,772)
Lowest Q (n=1,473)
HR changing patterns
G1: No change
G2: Changed to lower Q
G3: Changed to higher Q
Subgroups in the changed groups
G2-1: Down to lowest Q
G2-2: Down to 2nd or 3rd Q
G3-1: Up to 2nd or 3rd Q
G3-2: Up to highest Q
Subgroups in the no-change group
G1-1: Lowest to lowest Q
G1-2: 2nd to 2nd Q
G1-3: 3rd to 3rd Q
G1-4: Highest to highest Q

Figure S2

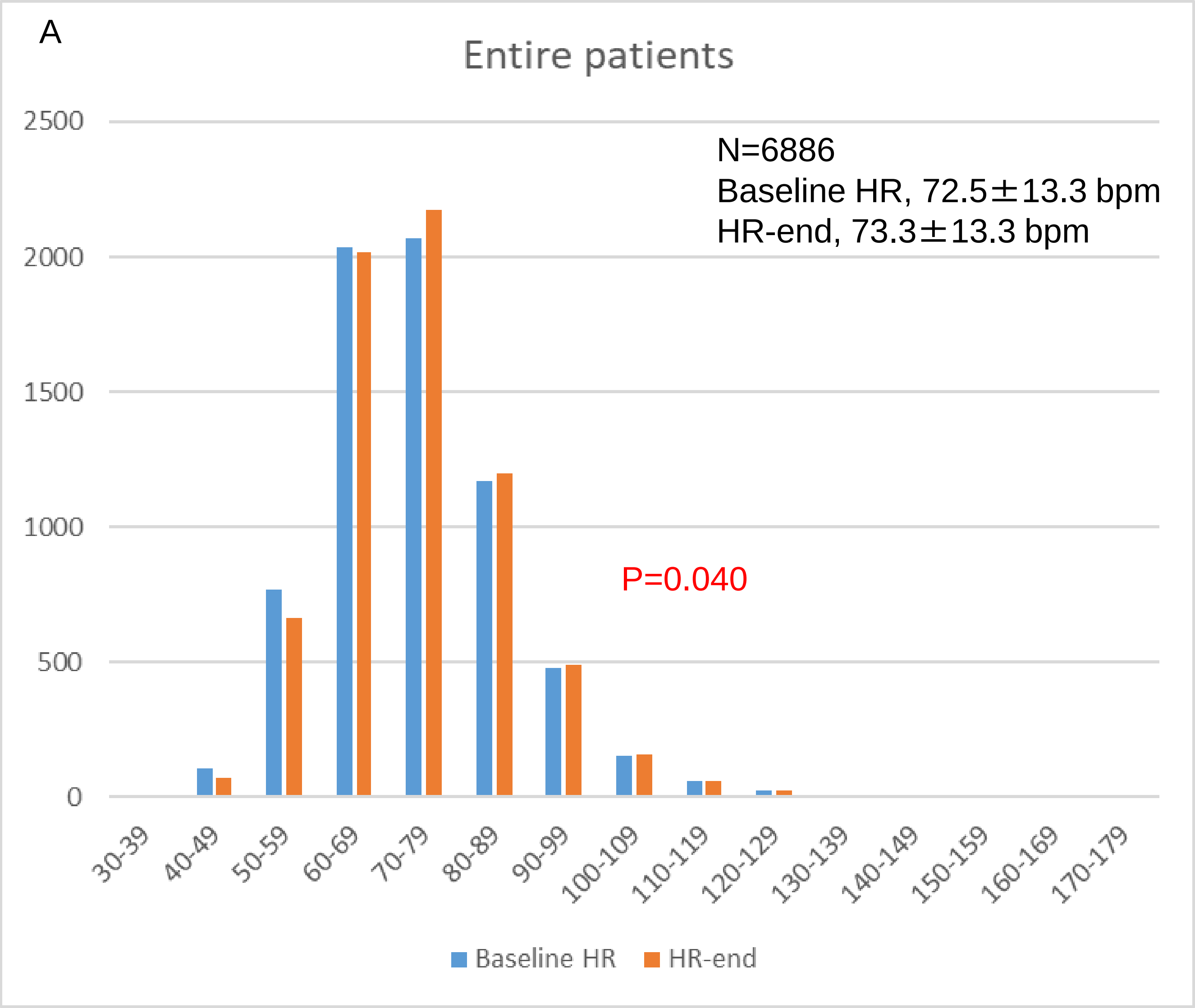
A
Entire patients
2500
2000
1500
1000
500
0
N=6886
Baseline HR, 72.5±13.3 bpm
HR-end, 73.3±13.3 bpm
P=0.040
30-39
40-49
50-59
60-69
70-79
80-89
90-99
100-109
110-119
120-129
130-139
140-149
150-159
160-169
170-179
Baseline HR
HR-end

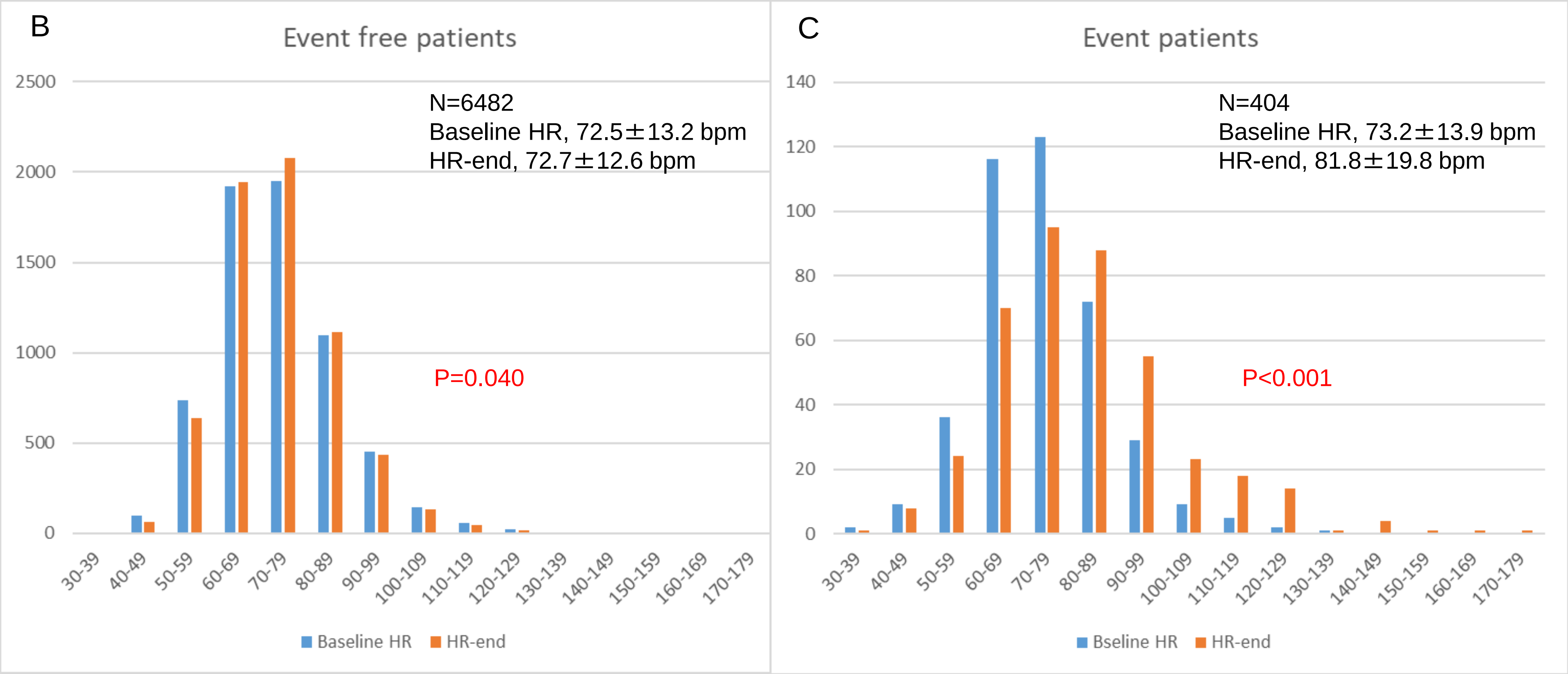

B
Event free patients
N=6482
Baseline HR, 72.5±13.2 bpm
HR-end, 72.7±12.6 bpm
P=0.040
2500
2000
1500
1000
500
0
30-39
40-49
50-59
60-69
70-79
80-89
90-99
100-109
110-119
120-129
130-139
140-149
150-159
160-169
170-179
Baseline HR
HR-end
C
Event patients
N=404
Baseline HR, 73.2±13.9 bpm
HR-end, 81.8±19.8 bpm
P<0.001
140
120
100
80
60
40
20
0
Bseline HR
HR-end

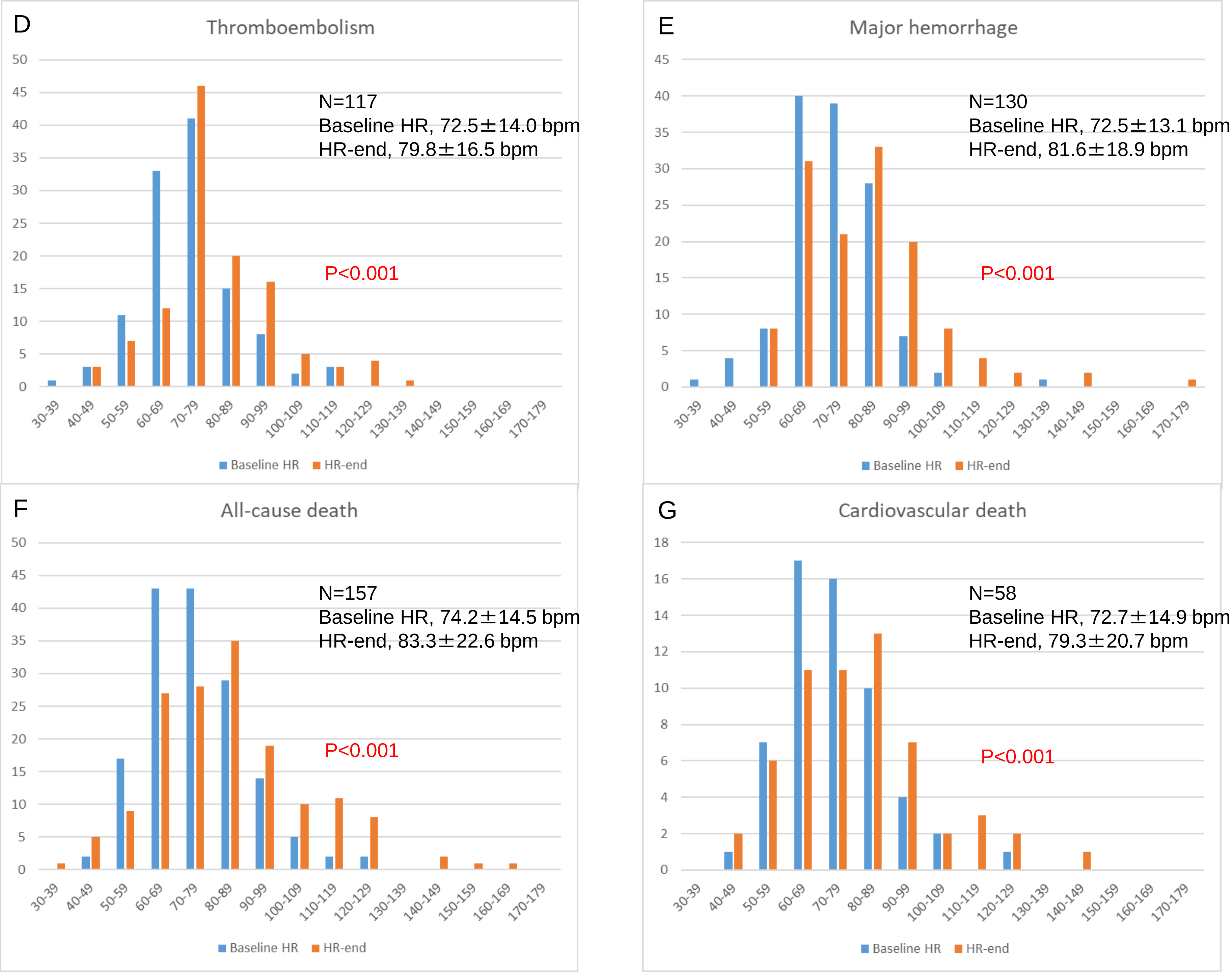
D
Thromboembolism
N=117
Baseline HR, 72.5±14.0 bpm
HR-end, 79.8±16.5 bpm
P<0.001
0 5 10 15 20 25 30 35 40 45 50
30-39 40-49 50-59 60-69 70-79 80-89 90-99 100-109 110-119 120-129 130-139 140-149 150-159 160-169 170-179
Baseline HR HR-end
E
Major hemorrhage
N=130
Baseline HR, 72.5±13.1 bpm
HR-end, 81.6±18.9 bpm
P<0.001
0 5 10 15 20 25 30 35 40 45
30-39 40-49 50-59 60-69 70-79 80-89 90-99 100-109 110-119 120-129 130-139 140-149 150-159 160-169 170-179
Baseline HR HR-end
F
All-cause death
N=157
Baseline HR, 74.2±14.5 bpm
HR-end, 83.3±22.6 bpm
P<0.001
0 5 10 15 20 25 30 35 40 45 50
30-39 40-49 50-59 60-69 70-79 80-89 90-99 100-109 110-119 120-129 130-139 140-149 150-159 160-169 170-179
Baseline HR HR-end
G
Cardiovascular death
N=58
Baseline HR, 72.7±14.9 bpm
HR-end, 79.3±20.7 bpm
P<0.001
0 2 4 6 8 10 12 14 16 18
30-39 40-49 50-59 60-69 70-79 80-89 90-99 100-109 110-119 120-129 130-139 140-149 150-159 160-169 170-179
Baseline HR HR-end

Figure S3

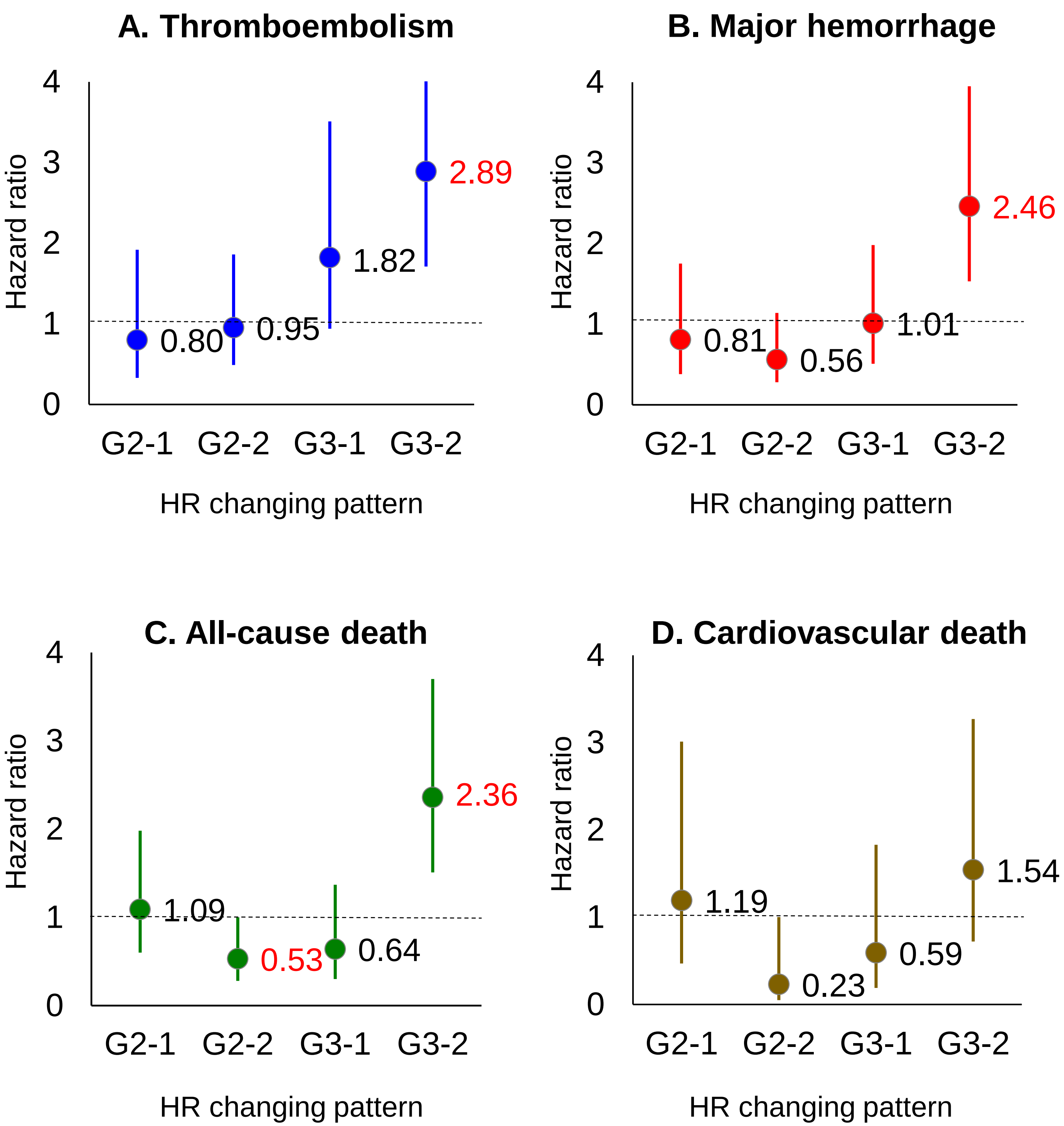
A. Thromboembolism
B. Major hemorrhage
C. All-cause death
D. Cardiovascular death
Hazard ratio
0
1
2
3
4
0.80
0.95
1.82
2.89
0.81
0.56
1.01
2.46
1.09
0.53
0.64
2.36
1.19
0.23
0.59
1.54
G2-1
G2-2
G3-1
G3-2
HR changing pattern

Figure S4

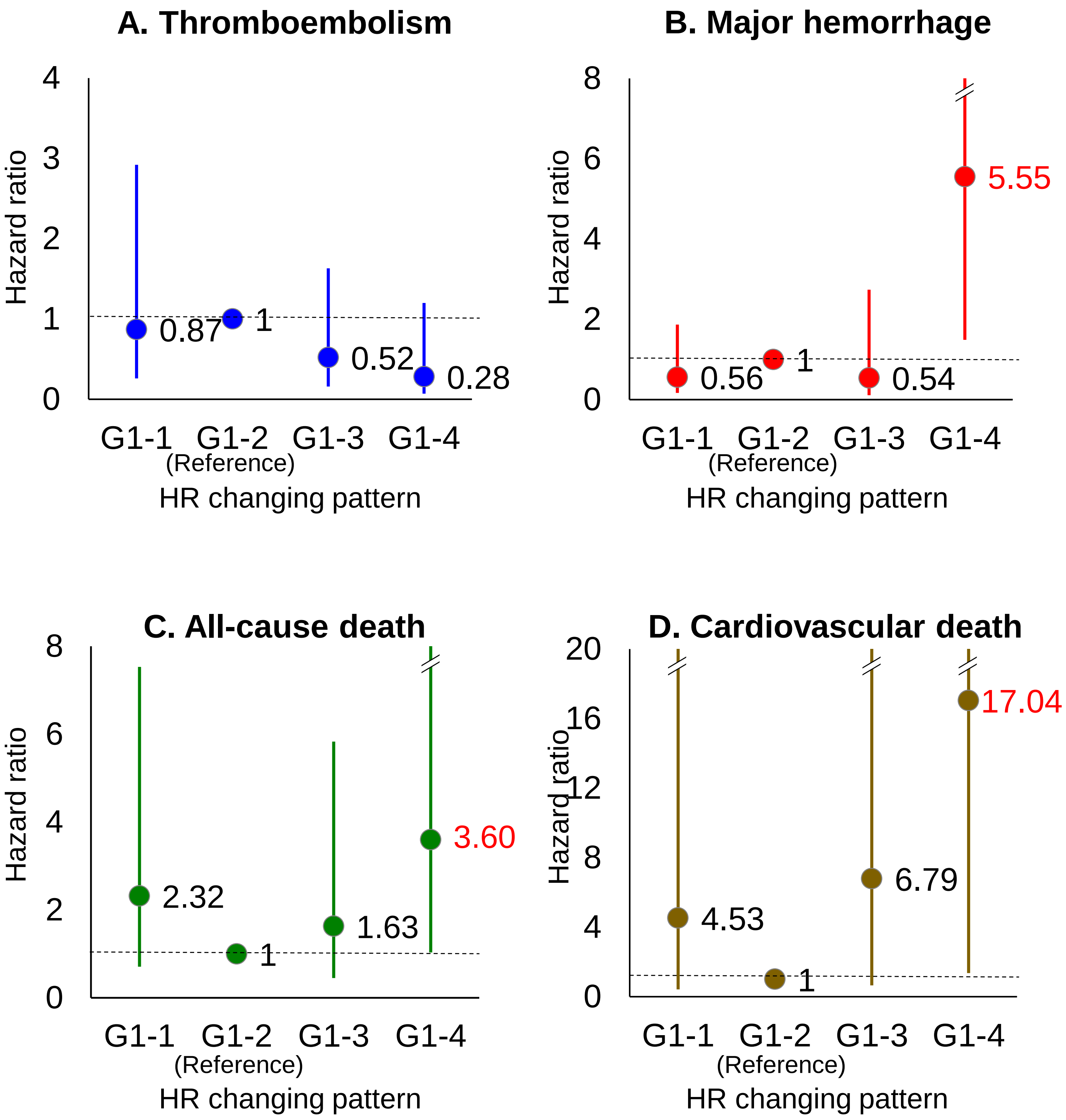

A. Thromboembolism
Hazard ratio
4
3
2
1
0
0.87
1
0.52
0.28
G1-1
G1-2
(Reference)
G1-3
G1-4
HR changing pattern
B. Major hemorrhage
Hazard ratio
8
6
4
2
0
0.56
1
0.54
5.55
G1-1
G1-2
(Reference)
G1-3
G1-4
HR changing pattern
C. All-cause death
Hazard ratio
8
6
4
2
0
2.32
1
1.63
3.60
G1-1
G1-2
(Reference)
G1-3
G1-4
HR changing pattern
D. Cardiovascular death
Hazard ratio
20
16
12
8
4
0
4.53
1
6.79
17.04
G1-1
G1-2
(Reference)
G1-3
G1-4
HR changing pattern

Figure S5

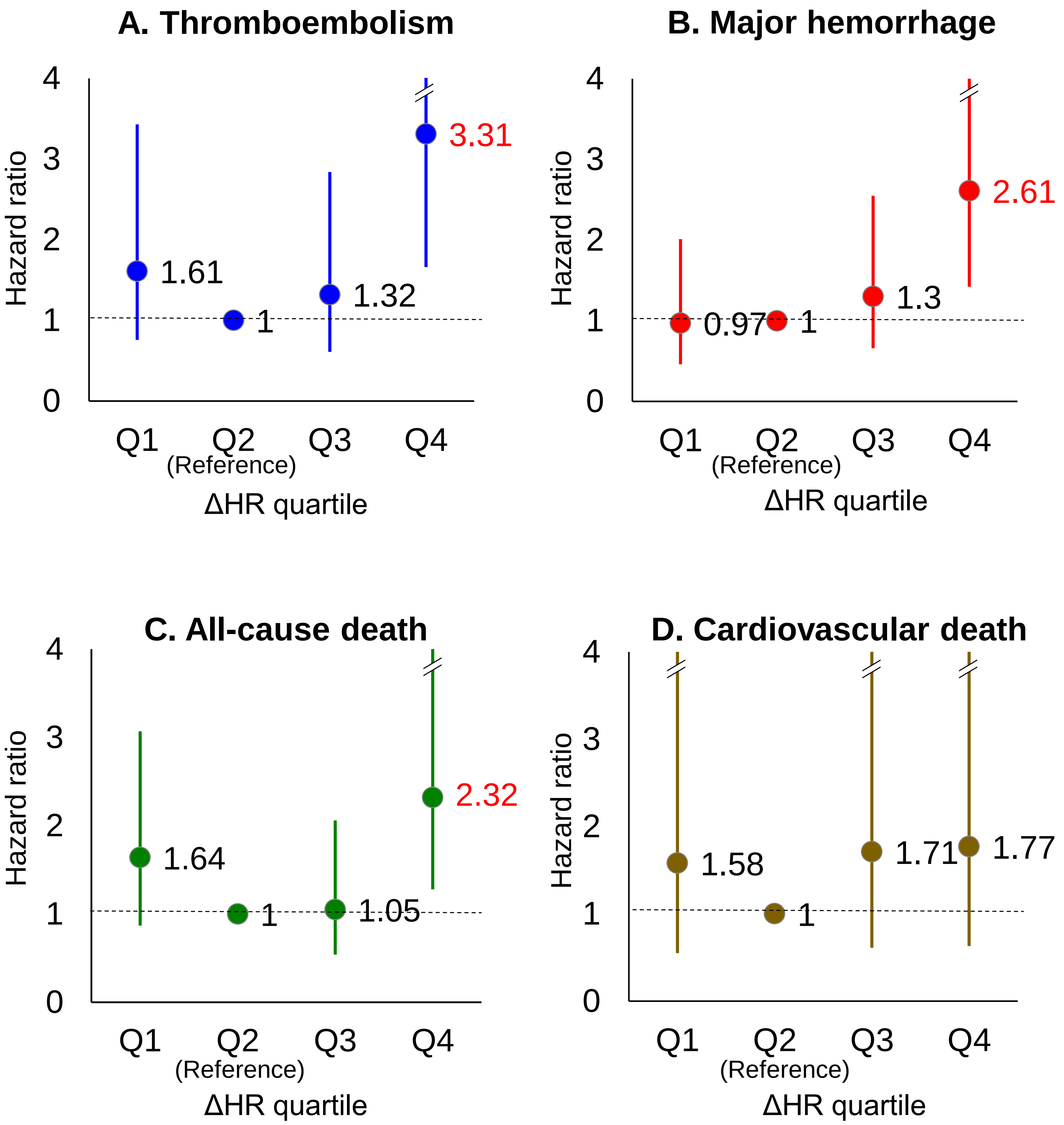
A. Thromboembolism
Hazard ratio
4
3
2
1
0
1.61
1
1.32
3.31
Q1
Q2
(Reference)
Q3
Q4
ΔHR quartile
B. Major hemorrhage
Hazard ratio
0.97
1
1.3
2.61
ΔHR quartile
C. All-cause death
Hazard ratio
1.64
1
1.05
2.32
ΔHR quartile
D. Cardiovascular death
Hazard ratio
1.58
1
1.71
1.77
ΔHR quartile

Supplementary Figure Legends

Figure S1. Changing patterns of heart rate quartiles and grouping

Bold arrows show groups of the changed to highest or lowest quartiles.

HR, heart rate; HR-end, heart rate at the time closest to an event or at the last visit of follow-up; Q, quartile; G, group.

Figure S2. Distribution of baseline HR and HR-end by event types for entire patients (A), event free patents (B), event patients (C), thromboembolism (D), major hemorrhage (E), all-cause death (F), and cardiovascular death (G)

HR, heart rate; HR-end, heart rate at the time closest to an event or at the last visit of follow-up; bpm, beats per minute.

P-values, comparison between baseline HR and HR-end by chi-square test.

Figure S3. Hazard ratios for thromboembolism (A), major hemorrhage (B), all-cause death (C), and cardiovascular death (D) by changing patterns of heart rate quartiles in changed groups

G, group; G2-1, Down to lowest quartile; G2-2, Down to 2nd or 3rd quartile; G3-1, Up to 2nd or 3rd quartile; G3-2: Up to highest quartile; HR, heart rate.

Hazard ratios were adjusted for components of the CHA_2DS_2-VASc score (congestive heart failure, hypertension, age ≥75 years, diabetes mellitus, history of stroke or TIA, vascular disease [coronary artery disease], age 65–74 years, female sex), warfarin and antiplatelet use, AF type, baseline systolic blood pressure and heart rate, creatinine

clearance, hemoglobin level, and β-blocker, K channel blocker, Ca channel blocker, and digitalis use.

Figure S4. Hazard ratios for thromboembolism (A), major hemorrhage (B), all-cause death (C), and cardiovascular death (D) by changing patterns of heart rate quartiles in no-change groups

G, group; G1-1, lowest to lowest quartile; G1-2, 2nd to 2nd quartile; G1-3, 3rd to 3rd quartile; G1-4, highest to highest quartile.

Hazard ratios were adjusted for variables as shown in Figure S3.

Figure S5. Hazard ratios for thromboembolism (A), major hemorrhage (B), all-cause death (C), and cardiovascular death (D) by Δheart rate quartiles

HR, heart rate; Q, quartile; Q1, <-7 bpm; Q2, -7 to -1 bpm; Q3, 0 to 7 bpm; Q4, ≥8 bpm; bpm, beats per minute.

Hazard ratios were adjusted for variables as shown in Figure S3.
